# Differences in maternal acceptance of non-national immunization program vaccines between single-child and multiple-children households a hospital-based cross-sectional study

**DOI:** 10.3389/fpubh.2026.1835253

**Published:** 2026-08-06

**Authors:** Rong Xing, Yuanling Wu, Yanli Lin, Yuting Hao, Liping Zhang, Manxia Hu, Ying Bai, Junyan Zhang, Fang Gao

**Affiliations:** 1 Department of Preventive Healthcare, Shanxi Bethune Hospital, Shanxi Academy of Medical Sciences, Tongji Shanxi Hospital, Third Hospital of Shanxi Medical University, Taiyuan, China; 2 Department of Clinical Laboratory, Shanxi Bethune Hospital, Shanxi Academy of Medical Sciences, Tongji Shanxi Hospital, Third Hospital of Shanxi Medical University, Taiyuan, China; 3 Department of Obstetrics and Gynecology, Shanxi Bethune Hospital, Shanxi Academy of Medical Sciences, Tongji Shanxi Hospital, Third Hospital of Shanxi Medical University, Taiyuan, China; 4 Early Childhood Education Center, Shanxi Provincial Children's Hospital, Taiyuan, China; 5 Department of Preventive Healthcare, Xiaodian Community Health Service Center, Taiyuan, China; 6 Department of Pediatrics, Shanxi Bethune Hospital, Shanxi Academy of Medical Sciences, Tongji Shanxi Hospital, Third Hospital of Shanxi Medical University, Taiyuan, China; 7 Department of Clinical Epidemiology and Evidence-Based Medicine, Shanxi Academy of Medical Sciences, Tongji Shanxi Hospital, Third Hospital of Shanxi Medical University, Taiyuan, China

**Keywords:** health service utilization, household structure, maternal willingness, non-national immunization program vaccines, preventive care decision-making

## Abstract

**Background:**

Non-National Immunization Program vaccines (non-NIPV) are recommended but not publicly funded in China, complementing National Immunization Program vaccines (NIPV) by providing protection against additional vaccine-preventable diseases. Their uptake depends largely on parental decision-making.

**Objectives:**

Assess maternal willingness to use non-NIPV (WNV) for their children across single-child households (SCH) and multiple-children households (MCH).

**Methods:**

A cross-sectional questionnaire survey was conducted among biological mothers of children aged 42 days to 3 years. The primary endpoint was the proportion expressing WNV. The exploratory endpoint compared WNV between household types. Multivariable logistic regression was used to estimate odds ratios (ORs) and 95% confidence intervals (CI). Propensity score overlap weighting was implemented as a sensitivity analysis.

**Results:**

Among 395 biological mothers of children aged 42 days to 3 years, mothers in SCH were younger than those in MCH (31.36 ± 4.24 vs. 35.51 ± 4.28 years). Compared with MCH, SCH had higher proportions of mothers with senior high school education or above (94.27% vs. 70.68%), employment (89.69% vs. 78.20%), and urban residence (83.97% vs. 72.93%). Overall WNV was 64.81%. WNV was higher in SCH than in MCH (67.94% vs. 58.65%). MCH was associated with lower odds of WNV (OR 0.34, 95% CI 0.18, 0.64, *p* = 0.001). Findings were consistent in overlap-weighted dataset analyses (OR 0.46, 95% CI 0.23, 0.90, *p* = 0.022).

**Conclusion:**

Maternal WNV varied by household structure, with lower willingness among MCH. Family composition may represent an important contextual factor in discretionary vaccination decisions. These findings support the consideration of household context and parental perceptions when designing communication strategies for non-NIPV.

## Introduction

Vaccination remains one of the most effective public health interventions for reducing childhood morbidity and mortality worldwide (1–3). While national immunization programs (NIP) ensure equitable access to essential vaccines, non-NIP vaccines (non-NIPV), which are recommended but not publicly funded, play an increasingly important role in preventing additional vaccine-preventable diseases across many health systems (4, 5).

Unlike National Immunization Program vaccines (NIPV), which are publicly funded, non-National Immunization Program vaccines (non-NIPV) are recommended but require out-of-pocket payment by parents, including vaccines against pneumococcal disease, varicella, rotavirus infection, human papillomavirus, enterovirus 71, and other vaccine-preventable diseases. Although vaccines included in the NIP have substantially reduced the burden of major childhood infectious diseases, non-NIPV provide additional protection against vaccine—preventable diseases that are not fully covered by the routine schedule, which may motivate some parents to seek non-NIPV despite the availability of NIPV. Previous studies have demonstrated that non-NIPV provide substantial public health benefits by reducing disease burden, preventing severe complications, and decreasing healthcare utilization (4, 6). Accordingly, a growing body of research has examined factors influencing parental acceptance of non-NIPV, including education, socioeconomic status, healthcare provider recommendations, and vaccine confidence (7–11).

Family composition, particularly the number of children within a household, represents an underexplored yet potentially influential determinant of parental vaccination behavior. From a life-course and behavioral economics perspective, parents with multiple children may approach health-related decisions differently from those with a single child, balancing accumulated caregiving experience, perceived disease risk, and financial and logistical constraints across siblings (12, 13). In contrast, parents of a single child may exhibit heightened risk aversion and stronger preventive orientation (14), *which may shape* their vaccination priorities when vaccines are not fully subsidized.

Despite these established benefits, uptake remains suboptimal because vaccination is voluntary and requires out-of-pocket payment, making parental willingness a critical determinant of vaccine uptake. Although parental vaccine decision-making is increasingly recognized as heterogeneous, whether family composition influences maternal willingness toward non-NIPV remains insufficiently understood, particularly in middle-income countries undergoing rapid demographic transitions.

As many countries, including China, experience shifts in fertility patterns and family size distributions, understanding how maternal decision-making differs between single-child households (SCH) and multiple-children households (MCH) has become increasingly relevant for optimizing vaccine communication strategies and improving coverage of non-NIPV (15, 16).

In this context, this study aimed to examine whether maternal willingness to use non-NIPV (WNV) for their children differs by family composition, with a particular focus on comparisons between mothers with a single child and those with multiple children.

## Methods

### Study design

This research employed a quantitative cross-sectional study, in which researchers conducted face-to-face questionnaire surveys with participants, and the questionnaires were self-administered by the participants.

### Participants

Participants were eligible if they met all of the following criteria: 1). Biological mothers aged 18 years or older with a child aged between 42 days and 3 years; 2). Attendance at routine child healthcare outpatient services at the Department of Preventive Healthcare of the study Hospital; 3). Mothers who served as the primary caregivers or the principal decision-makers regarding their child’s vaccination; 4). Voluntary participation with provision of written informed consent.

Participants were excluded if any of the following criteria were met: 1). The child had congenital or genetic diseases, or primary immunodeficiency disorders; 2). The mother had difficulties in comprehension, communication, or cooperation that precluded completion of the questionnaire or interview; 3). The child had already received all non-NIPV, making it impossible to assess future willingness to vaccinate with this category of vaccines; 4). The mother or the child was participating in other clinical studies that could influence vaccination decision-making.

In the child healthcare clinics where recruitment was conducted, mothers were typically the accompanying caregivers. Therefore, eligible biological mothers were approached for recruitment, and study staff verbally confirmed that each participant met the eligibility criterion of being the child’s primary caregiver or principal decision-maker regarding vaccination before enrollment.

Participants were consecutively recruited from the Department of Preventive Healthcare of the study hospital (Shanxi Bethune Hospital, Taiyuan, China) during routine child healthcare visits or vaccination appointments. Eligible biological mothers were invited to complete the questionnaire voluntarily while attending these outpatient services.

### Questionnaire

The questionnaire was developed by the investigators based on previous vaccine acceptance studies and the clinical context of non-National Immunization Program vaccine decision-making in China. The survey mainly included structured factual and preference-based items regarding demographic characteristics, household composition, vaccination history, awareness of non-National Immunization Program vaccines, perceived importance and safety of vaccination, and willingness to vaccinate. Because the questionnaire was designed as a study-specific structured survey rather than a psychometric scale intended to measure latent psychological constructs, formal reliability and validity testing were not performed. Detailed information on the questionnaire, including all survey items and response options, is provided in the Appendix. Because the questionnaire primarily collected factual information and single-item perceptions rather than measuring latent psychological constructs, formal psychometric reliability and validity testing was not applicable.

### Grouping

Participants were categorized according to the number of children in the household into two groups: SCH and MCH, with MCH defined as families with two or more children.

### Data collection

Data were collected on site at the child healthcare outpatient clinic of the Department of Preventive Healthcare of the study Hospital. Researchers were present during data collection, and participants completed a structured questionnaire independently. The questionnaire captured maternal and household sociodemographic characteristics, knowledge and attitudes toward non-NIPV, previous uptake of non-NIPV, and willingness to receive such vaccines in the future.

### Study endpoints

The primary outcome was maternal willingness to vaccinate with non-NIPV. Maternal willingness to vaccinate with non-NIPV was assessed using the question: “Are you willing to vaccinate your child with non-NIPV?” Response options included “Yes,” “No,” and “Uncertain.” For the primary analysis, “No” and “Uncertain” responses were classified as unwillingness. For regression analyses, categorical predictors derived from Likert-scale items were dichotomized. Detailed questionnaire items, response options, and coding rules are provided in Supplementary sTable S1 in the Appendix. Exploratory endpoint was comparison of maternal WNV for their children between SCH and MCH.

### Statistical analysis

The analysis presented continuous variables as the mean with standard deviation (SD) for normally distributed data, or as the median with interquartile range (IQR) presented as the 1st quartile to the 3rd quartile (Q1-Q3) for non-normally distributed data. The Student t-test was applied for normally distributed data, while the Mann–Whitney test was employed for data with non-normal distributions. Categorical variables were represented as counts and percentages. The statistical significance of categorical variables was assessed using Pearson’s chi-square test or Fisher’s exact test.

For descriptive analyses, selected sociodemographic variables were dichotomized prior to analysis. Educational attainment was categorized as senior high school or above versus below senior high school. Occupation was classified as working mother versus full-time homemaker, residential area as urban versus rural, and mode of delivery as cesarean versus vaginal delivery. Variables included in the regression analyses were selected based on previous literature on vaccine acceptance, clinical relevance, and potential associations with maternal willingness to vaccinate. Potential confounders considered in the analyses included maternal age, education level, occupation, residential area, vaccination history, awareness of non-National Immunization Program vaccines, and perceptions regarding vaccine importance, safety, effectiveness, and serious adverse effects.

The exploratory endpoint was analyzed using multivariable logistic regression. Candidate predictors included individual-, interpersonal-, and questionnaire-derived variables related to knowledge, attitudes, previous vaccination experience, affordability, healthcare professionals’ recommendations, and delivery mode. A backward stepwise selection procedure based on Wald tests was applied, with variables sequentially removed when their *p* values were ≥0.10 (17) until the final model was obtained.

Model coefficients were re-estimated after each step, and the procedure continued until no variable met the removal criterion. Group status (SCH vs. MCH) was forced into all models regardless of statistical significance. Results are presented as adjusted odds ratios (ORs) with 95% CIs. Additionally, propensity score overlap weighting was applied to balance sociodemographic characteristics between the two groups, and the resulting *overlap-weighted dataset* was used to perform a sensitivity analysis of the primary endpoint.

Stata SE 13, R software (version 4.4.3), and GraphPad (8.0.2) were applied for the data analysis.

### Sample size calculation

Sample size of the first phase was estimated on the primary endpoint, defined as the proportion of mothers willing to vaccinate their child with non-NIPV in the future. Based on the pilot survey, this proportion was estimated to be approximately 65% (*p* = 0.65). With a two-sided *α* = 0.05 and a desired margin of error of ±5% for the 95% CI, the required sample size was calculated using the single-proportion formula (*n* = Z^2^ × *p* × (1—*p*)/*d*^2^), where *Z* = 1.96 and d = 0.05. This yielded an estimated sample size of approximately 350 participants. To account for potential non-response or invalid questionnaires, a compensation factor of 1.2 was applied, resulting in a minimum required sample size of 420 participants.

## Results

**Figure 1:**
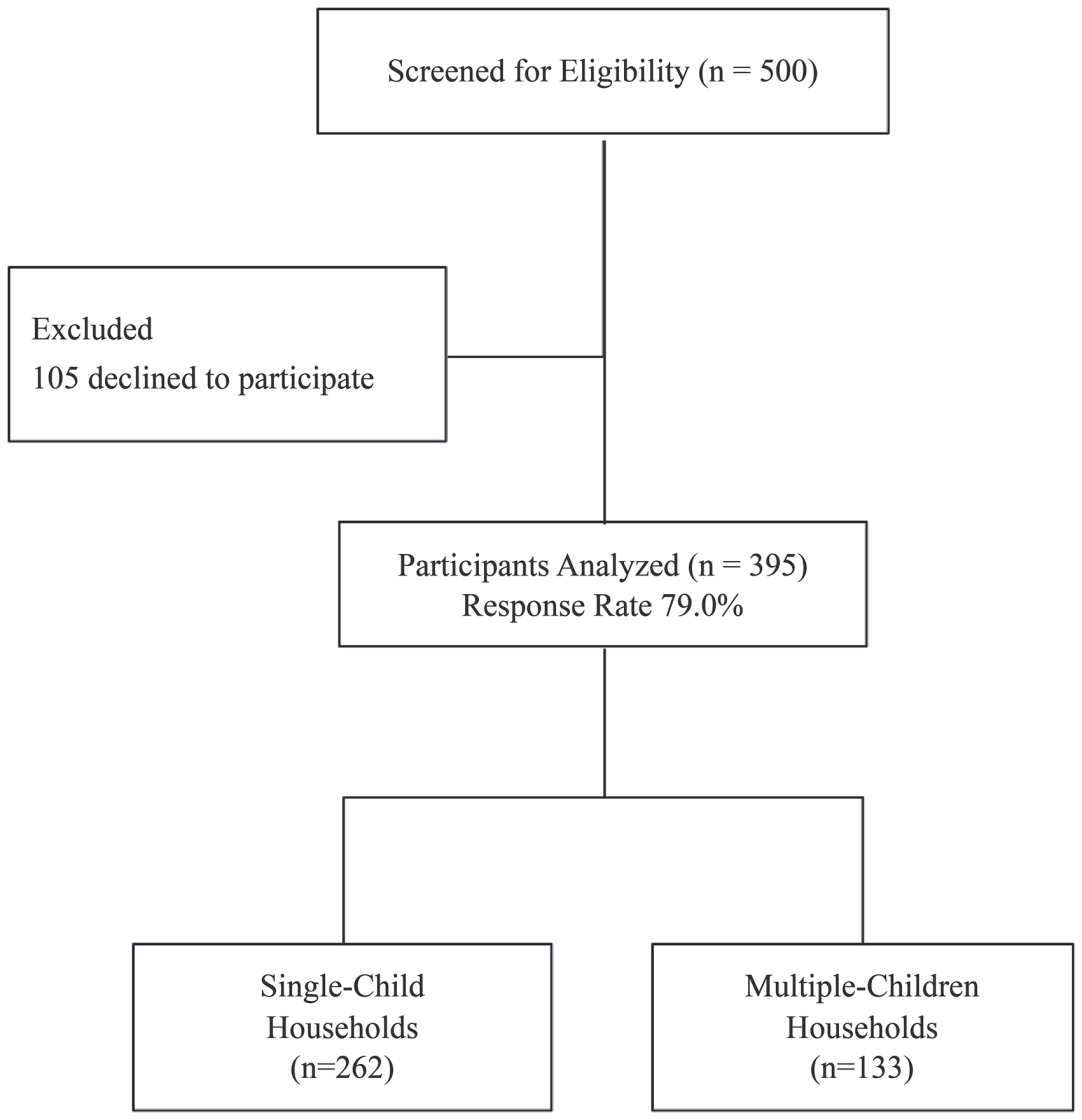
Flow diagram of participants.

The study was conducted between September 2025 and January 2026. Of the 500 eligible participants invited to participate, 105 declined, resulting in 395 completed questionnaires and a response rate of 79.0%. Ultimately, 500 eligible participants were screened and 395 valid questionnaires were completed and included in the final analysis (Figure 1).

### Sociodemographic characteristics of participants

In the original dataset, mothers in SCH were younger than those in MCH (31.36 (4.24) vs. 35.51 (4.28), *p* < 0.001). The proportion of participants with SHS or above was higher in SCH than in MCH (94.27% vs. 70.68%, *p* < 0.001). A higher proportion of working mothers was observed in SCH compared with MCH (89.69% vs. 78.20%, *p* = 0.003). The proportion of participants residing in urban areas was also higher in SCH than in MCH (83.97% vs. 72.93%, *p* = 0.013). Monthly income of at least 10,000 CNY and mode of delivery were similar between groups. In the overlap-weighted dataset, baseline sociodemographic characteristics were well balanced between SCH and MCH, with all SMD < 0.001 and all *p*-values >0.999 (Table 1).

**Table 1:** Sociodemographic characteristics of participants in the original and overlap-weighted datasets.

Characteristics	Original dataset				Overlap-weighted dataset			
	SCH (*n* = 262)	MCH (*n* = 133)	SMD	*p*-value	SCH (*n* = 66.13)	MCH (*n* = 66.13)	SMD	*p*-value
Age [mean (SD)]	31.36 (4.24)	35.51 (4.28)	0.974	<0.001	33.93 (4.76)	33.93 (4.04)	<0.001	>0.999
Education (SHS above)	247 (94.27)	94 (70.68)	0.653	<0.001	57.76 (87.35)	57.76 (87.35)	<0.001	>0.999
Occupation (Working mother)	235 (89.69)	104 (78.20)	0.371	0.003	57.15 (86.43)	57.15 (86.43)	<0.001	>0.999
Monthly income at least 10 K CNY	68 (25.95)	23 (17.29)	0.212	0.071	15.01 (22.70)	15.01 (22.70)	<0.001	>0.999
Residential area (Urban area)	220 (83.97)	97 (72.93)	0.271	0.013	53.35 (80.68)	53.35 (80.68)	<0.001	>0.999
Mode of delivery (Cesarean)	140 (53.44)	65 (48.87)	0.091	0.453	32.79 (49.59)	32.79 (49.59)	<0.001	>0.999

SCH, Single-child households; MCH, Multiple-children households; SMD, Standardized mean difference; SD, Standard deviation; SHS, Senior high school; CNY, Chinese Yuan.

Values in parentheses represent percentages unless otherwise specified. Educational attainment was categorized as SHS or above versus SHS or below. Mode of delivery was classified as cesarean versus vaginal delivery. Occupation was categorized as working mother versus full-time homemaker. Residential area was classified as urban versus rural.

### Participants’ responses to key questionnaire items

In the original dataset, the proportion of participants reporting concern about serious adverse effects of non-NIPV was higher in SCH than in MCH (77.48% vs. 58.65%, *p* < 0.001). The proportion of participants perceiving healthcare professionals’ recommendations as influential in the choice of non-NIPV was also higher in SCH than in MCH (97.71% vs. 88.72%, *p* < 0.001). In addition, a lower proportion of children in SCH had ever received non-NIPV compared with MCH (47.71% vs. 69.92%, *p* < 0.001). No differences were observed between SCH and MCH with respect to awareness of the difference between NIP and non-NIPV, perceived importance of non-NIPV, perceived safety of non-NIPV, perceived effectiveness of non-NIPV in preventing disease, perceived price reasonableness of non-NIPV, or WNV. In the overlap-weighted dataset, group differences across questionnaire items were largely attenuated, with small SMD and no statistically significant differences observed between SCH and MCH (Supplementary sTable S2 in the Appendix).

### Primary endpoint

Table 2 presents the overall proportion of mothers who expressed WNV. In the full sample, the WNV was 64.81% (95% CI 59.95, 69.38). When summarized by household type, the WNV was 67.94% (95% CI 62.02, 73.34) among SCH and 58.65% (95% CI 50.05, 66.75) among MCH.

**Table 2:** The rate of willingness to use Non-NIPV.

Outcome	All (*n* = 395)	SCH (*n* = 262)	MCH (*n* = 133)
Willingness to use non-NIPV, % (95% CI)	64.81(59.95, 69.38)	67.94 (62.02, 73.34)	58.65 (50.05, 66.75)

SCH, Single-child households; MCH, Multiple-children households; NIPV, National immunization program vaccine; WNV, willingness to use non-NIPV; CI, Confidence interval.

### Exploratory endpoint

In the original dataset, perceived non-NIPV safety was associated with higher odds of WNV (OR 3.33 (95% CI 1.64, 6.78), *p* = 0.001). Perceived non-NIPV importance was also associated with higher odds of WNV (OR 4.67 (95% CI 2.15, 10.12), *p* < 0.001). Cesarean delivery was associated with lower odds of WNV (OR 0.48 (95% CI 0.27, 0.86), *p* = 0.013). Child ever received non-NIPV was associated with higher odds of WNV (OR 4.50 (95% CI 2.38, 8.51), *p* < 0.001). Awareness of the difference between NIPV and non-NIPV was associated with lower odds of WNV (OR 0.38 (95% CI 0.19, 0.73), *p* = 0.004). Perceived effectiveness of non-NIPV in preventing disease was associated with higher odds of WNV (OR 3.38 (95% CI 1.83, 6.23), *p* < 0.001). Perceived price reasonableness of non-NIPV was associated with higher odds of WNV (OR 1.98 (95% CI 1.06, 3.70), *p* = 0.031). Compared with SCH, MCH was associated with lower odds of WNV (OR 0.34 (95% CI 0.18, 0.64), *p* = 0.001). In the overlap-weighted dataset, results were consistent with the primary analysis. Cesarean delivery was not retained in the sensitivity analysis after covariate balancing (Table 3).

**Table 3:** Multivariable logistic regression analysis of the exploratory endpoint.

Variables	Original dataset (primary analysis)		Overlap-weighted dataset (sensitivity analysis)	
	OR (95% CI)	*p*-value	OR (95% CI)	*p*-value
non-NIPV safety	3.33 (1.64, 6.78)	0.001	3.37 (1.48, 7.70)	0.004
non-NIPV importance	4.67 (2.15, 10.12)	<0.001	4.30 (1.68, 10.96)	0.002
Cesarean delivery	0.48 (0.27, 0.86)	0.013	—	—
Child ever received non-NIPV	4.50 (2.38, 8.51)	<0.001	5.85 (2.91, 11.73)	<0.001
Awareness of the difference between NIPV and non-NIPV	0.38 (0.19, 0.73)	0.004	0.32 (0.15, 0.67)	0.003
Effectiveness of non-NIPV in preventing disease	3.38 (1.83, 6.23)	<0.001	2.97 (1.44, 6.12)	0.003
Price reasonableness of non-NIPV	1.98 (1.06, 3.70)	0.031	2.20 (1.05, 4.61)	0.038
MCH	0.34 (0.18, 0.64)	0.001	0.46 (0.23, 0.90)	0.022

OR, Odds ratio; CI, Confidence interval; NIPV, National immunization program vaccine; MCH, Multiple-children households.

## Discussion

### Summary of key findings

Parental acceptance of non-NIPV has become an increasingly important issue in childhood immunization programs in settings where such vaccines are recommended but not publicly funded. In such contexts, vaccination decisions are often closely shaped by out-of-pocket costs and the lack of insurance reimbursement (18, 19). Understanding maternal decision-making regarding non-NIPV is therefore essential in contexts where vaccination choices are strongly shaped by family-level considerations and out-of-pocket payment requirements (20, 21). In this study, approximately two-thirds of mothers expressed willingness to vaccinate their children with non-NIPV, reflecting a moderate level of acceptance in this clinical setting. Notably, the present study demonstrated that maternal WNV was significantly lower among MCH than among SCH. Although previous studies have increasingly recognized that parental vaccination decisions are influenced by family characteristics, direct comparisons between single-child and multiple-children households have remained limited. Our findings extend the existing literature by demonstrating an independent association between household structure and maternal willingness to vaccinate with non-NIPV. This association remained evident after adjustment for sociodemographic characteristics and vaccination-related perceptions in multivariable logistic regression, and was further supported by analyses using an overlap-weighted dataset to balance baseline characteristics between groups. This difference remained evident after adjustment for sociodemographic characteristics and vaccination-related perceptions in multivariable logistic regression, and was further supported by analyses using an overlap-weighted dataset to balance baseline characteristics between groups. The consistency of findings across analytic approaches underscores the robustness of the association between household structure and maternal WNV, beyond confounding by measured background factors. This pattern suggests that household structure may function not merely as a proxy for socioeconomic differences but as an independent contextual factor influencing discretionary vaccination decisions.

### Household structure and vaccination willingness

Family composition in terms of the number of children may serve as a proxy for differences in resource allocation and risk perception within families, both of which are central to vaccination decision-making regarding non-NIPV (22, 23). Mothers from MCH may face greater cumulative financial constraints and competing health priorities across siblings, which may lead to more conservative decision-making regarding optional vaccines. At the same time, prior caregiving and vaccination experience accumulated across children may contribute to a recalibration of perceived disease risk and vaccine necessity, potentially reducing the urgency to pursue additional non-NIPV (24).

In contrast, SCH may adopt a more prevention-oriented approach, characterized by heightened risk aversion and a stronger willingness to invest in measures perceived (25). Such differences are unlikely to be as salient for vaccines included in routine immunization schedules, but may become more pronounced in the context of non-NIPV, where cost considerations, subjective risk–benefit assessments, and parental discretion exert a greater influence on uptake decisions. The observed disparity in WNV between household types in this study is therefore consistent with broader behavioral and life-course perspectives suggesting that family composition systematically shapes health-related choices under conditions of uncertainty and partial financial responsibility (26). Although these explanations are supported by previous literature, the underlying mechanisms were not directly assessed in the present study and therefore should be interpreted as hypotheses requiring further investigation.

### Determinants of WNV

While household structure provides the broader contextual background for vaccination decision-making, the present findings also indicate that maternal willingness is directly shaped by several cognitive and experiential determinants. The present findings highlight that maternal WNV is shaped by a set of interrelated cognitive and experiential factors rather than by household structure alone. Perceptions of vaccine importance, safety, and effectiveness emerged as key value-based determinants, underscoring the central role of parental judgment in weighing anticipated benefits against perceived risks when vaccination decisions are discretionary and require out-of-pocket payment. Previous studies have similarly suggested that these evaluative dimensions of perceived importance, safety, and effectiveness constitute the core of parental decision-making for optional childhood vaccines, particularly in contexts where formal policy endorsement is limited (27, 28).

Beyond value judgments, prior vaccination experience appears to exert a strong influence on subsequent willingness to vaccinate. Mothers whose children had previously received non-NIPV were more likely to express future willingness, consistent with a path-dependent decision process in which earlier acceptance reinforces trust, reduces uncertainty, and lowers psychological barriers to continued uptake (29). Such experiential effects may operate independently of family structure, but their impact can be amplified or attenuated depending on the broader caregiving context in which decisions are made.

An additional and noteworthy finding is the inverse association between awareness of the distinction between NIPV and non-NIPV and willingness to vaccinate. Rather than reflecting better-informed decision-making per se, heightened awareness may increase sensitivity to the institutional boundary between publicly funded and self-paid vaccines (30), thereby accentuating perceptions of optionality, uncertainty, or responsibility shifting to the family. It should be noted that awareness of the distinction between NIPV and non-NIPV likely reflects a composite understanding that extends beyond factual knowledge. Such awareness may be interpreted as recognition of differences in funding mechanisms and policy endorsement, and may also be accompanied by perceptions that vaccines not included in the National Immunization Program are less essential or supported by weaker evidence compared with those included in routine schedules. Heightened sensitivity to these institutional and evidentiary boundaries may therefore reinforce caution and reduce WNV, rather than facilitating uptake. Together, these determinants appear to function across different household types, while family structure itself provides a contextual backdrop that may magnify or buffer the influence of such cognitive and experiential factors on vaccination decisions (31).

### Implications for practice

The findings of this study suggest that efforts to promote non-NIPV should account for heterogeneity in parental decision-making rather than assuming uniform responses across families. Household context may influence how parents interpret vaccine-related information and weigh perceived benefits, risks, and financial considerations when making discretionary vaccination decisions.

In clinical practice, communication from healthcare professionals plays a central role in supporting parental decision-making regarding optional vaccines. Clear explanations of vaccine safety, effectiveness, and potential adverse reactions may help reduce uncertainty and facilitate more informed choices. In addition, structured communication regarding the distinction between vaccines included in the National Immunization Program and those outside the program may help parents better understand differences in funding mechanisms and policy positioning.

Together, these findings highlight the importance of transparent and context-sensitive communication strategies in discussions of non-NIPV in routine vaccination settings.

### Limitations

Several limitations should be acknowledged. First, the cross-sectional design precludes causal inference regarding the relationship between household structure and WNV. Second, this study was conducted at a single-center hospital-based outpatient clinic, which may limit the generalizability of the findings to mothers from other regions of China or to populations with different demographic characteristics or healthcare settings. Third, the use of self-administered questionnaires may introduce information bias related to recall or social desirability.

In addition, sociodemographic differences observed between SCH and MCH in the original sample likely reflect real-world family characteristics rather than sampling imbalance. Although an overlap-weighted approach was applied to improve comparability between groups and to assess the robustness of the findings, residual confounding cannot be fully excluded. Accordingly, the observed associations should be interpreted with caution and viewed as descriptive of patterns in parental decision-making rather than as evidence of causal effects.

## Conclusion

In summary, maternal WNV differed by household structure, with lower willingness observed among mothers from MCH. This association remained consistent across analytic approaches, suggesting that family composition represents a meaningful contextual factor in non-NIPV decision-making. Consideration of household context, together with key cognitive and experiential determinants, may support more refined clinical communication and engagement strategies when discussing non-NIPV. These findings may also inform policymakers in developing targeted communication and support strategies to promote equitable uptake of non-NIPV, particularly among multiple-children households. Given the single-center design, multicenter studies are warranted to confirm the generalizability of these findings.

## Data Availability

The raw data supporting the conclusions of this article will be made available by the authors, without undue reservation.
