# Supplementary material for: Differences in maternal acceptance of non-national immunization program vaccines between single-child and multiple-children households a hospital-based cross-sectional study
Source: Front Public Health. 2026 Aug 6;14:1835253. doi: 10.3389/fpubh.2026.1835253 (PMC13490105; doi:10.3389/fpubh.2026.1835253)
Supplement: Supplementary file 1 [file Supplementary_file_1.DOCX]

**Contents of the Appendix of Differences in Maternal Acceptance of
Non-National Immunization Program Vaccines Between
Single-Child and Multiple-Children Households**

[I.	Demographic Characteristics (omitted)	2](#_Toc233733976)

[II.	Vaccine Knowledge and Awareness	2](#_Toc233733977)

[III.	Attitudes and Beliefs Regarding Non-immunization Program Vaccines	2](#_Toc233733978)

[IV.	Previous Receipt of Non-immunization Program Vaccines	3](#_Toc233733979)

[V.	Future Willingness to Vaccinate and Influencing Factors	3](#_Toc233733980)

[VI.	Suggestions Regarding Policy and Services	4](#_Toc233733981)

[sTable 1. Questionnaire Variables, Response Options, and Coding for Regression Analyses	5](#_Toc233733982)

[sTable 2. Participants’ Responses to Key Questionnaire Items in the Original and Overlap-Weighted PSM Datasets	5](#_Toc233733983)

Questionnaire on Factors Influencing Mothers' Acceptance of Non-immunization Program Vaccination for Infants and Young Children

# I. Demographic Characteristics (omitted)

# II. Vaccine Knowledge and Awareness

## 1. Do you understand the difference between non-immunization program vaccines and immunization program vaccines?

□ Very well □ Relatively well □ Fairly □ Not very well □ Not at all

## 2. What are your main sources of vaccine-related knowledge? (Multiple responses allowed)

□ Explanations from hospital physicians □ Community health education □ Online platforms (e.g., WeChat, short-video platforms) □ Recommendations from relatives or friends □ Other: ______

## 3. How important do you think non-immunization program vaccines are?

□ Very important
□ Relatively important
□ Moderately important
□ Not very important
□ Not important at all

## 5. How safe do you think non-immunization program vaccines are?

□ Very safe □ Relatively safe □ Moderately safe □ Not very safe □ Not safe at all

# III. Attitudes and Beliefs Regarding Non-immunization Program Vaccines

## 6. Do you believe that non-immunization program vaccines can effectively prevent diseases?

□ Yes □ No □ Uncertain

## 7. Are you concerned that non-immunization program vaccines may cause serious adverse reactions?

□ Very concerned
□ Relatively concerned
□ Moderately concerned
□ Not very concerned
□ Not concerned at all

## 9. Do you think the prices of non-immunization program vaccines are reasonable?

□ Very reasonable
□ Relatively reasonable
□ Moderately reasonable
□ Not very reasonable
□ Not reasonable at all

## 11. To what extent do recommendations from doctors or healthcare workers influence your choice of non-immunization program vaccines?

□ A great deal □ To some extent □ Only slightly □ No influence

# IV. Previous Receipt of Non-immunization Program Vaccines

## 12. Has your child ever received any non-immunization program vaccine?

□ Yes □ No

### If yes, what were the main reasons for vaccination? (Multiple responses allowed)

□ Disease prevention
□ Doctor's recommendation
□ Other parents around me had their children vaccinated
□ The price was acceptable
□ Other: ______

## 13. If no, what were the main reasons for not vaccinating? (Multiple responses allowed)

□ Concern about safety
□ High price
□ Belief that it was unnecessary
□ Lack of relevant information
□ Other: ______

# VI. Future Willingness to Vaccinate and Influencing Factors

## 14. Would you be willing to have your child receive non-immunization program vaccines in the future?

□ Very willing □ Relatively willing □ Uncertain □ Not very willing □ Completely unwilling

## 15. If you are unwilling to vaccinate, what are the main reasons? (Multiple responses allowed)

□ Heavy financial burden
□ Concern about adverse reactions
□ Lack of trust
□ Belief that the disease risk is low
□ Other: ______

## 16. Which measures do you think could increase your willingness to vaccinate with non-immunization program vaccines? (Multiple responses allowed)

□ Lower prices
□ Strengthened communication on vaccine safety
□ Detailed explanations from doctors
□ Vaccination subsidies
□ Other: ______

## 17. Key factors influencing your decision-making (rank by importance):

[ ] Vaccine safety

[ ] Professional advice from doctors

[ ] Household economic conditions

[ ] Vaccine preventive effectiveness

[ ] Convenience of vaccination

[ ] Experiences of relatives and friends

## 19. Which type of institution would you prefer for your child's receipt of non-immunization program vaccines?

□ Public tertiary Grade A hospital
□ Community health service center
□ Private medical institution
□ Maternal and child health hospital
□ Other: ______

# VIII. Suggestions Regarding Policy and Services

## 20. What types of vaccine-related support would you like to receive? (Multiple responses allowed)

□ Government price subsidies □ Medical insurance coverage □ Health education lectures □ Green channel for vaccination appointments □ Adverse-reaction protection mechanism

## 21. Other suggestions or concerns:

____________________________________________________________

# sTable 1. Questionnaire Variables, Response Options, and Coding for Regression Analyses

| Variable | Questionnaire response options | Coding for regression |
| --- | --- | --- |
| Non-NIPV safety | Very safe / Relatively safe / Moderately safe / Not very safe / Not safe at all | Group 1: Moderately safe, not very safe, or not safe at all Group 2: Relatively safe or very safe |
| Non-NIPV importance | Very important / Relatively important / Moderately important / Not very important / Not important at all | Group 1: Moderately important, not very important, or not important at all  Group 2: Relatively important or very important |
| Awareness of the difference between NIPV and non-NIPV | Very well / Relatively well / Fairly / Not very well / Not at all | Group 1: Fairly, not very well, or not at all Group 2: Relatively well or very well |
| Effectiveness of non-NIPV in preventing disease | Yes / No / Uncertain | Group 1: No or uncertain Group 2: Yes |
| Price reasonableness of non-NIPV | Very reasonable / Relatively reasonable / Moderately reasonable / Not very reasonable / Not reasonable at all | Group 1: Moderately reasonable, not very reasonable, or not reasonable at all  Group 2: Relatively reasonable or very reasonable |
| Child ever received non-NIPV | Yes / No | Group 1: No Group 2: Yes |
| Cesarean delivery | Yes / No | Group 1: No Group 2: Yes |
| Variable | Questionnaire item | Coding for regression |
| Non-NIPV importance | Very important → Not important at all | Very/Relatively important =1; others =0 |
| Non-NIPV safety | Very safe → Not safe at all | Very/Relatively safe =1; others =0 |
| Effectiveness | Yes / No / Uncertain | Yes=1; No/Uncertain=0 |
| Price reasonableness | Very reasonable → Not reasonable at all | Very/Relatively reasonable=1; others=0 |
| Awareness | Very well → Not at all | Very/Relatively well=1; others=0 |
| Child ever received non-NIPV | Yes/No | Yes=1; No=0 |
| Cesarean delivery | Yes/No | Yes=1; No=0 |

NIPV: National Immunization Program Vaccine;

# sTable 2. Participants’ Responses to Key Questionnaire Items in the Original and Overlap-Weighted PSM Datasets

|  | Original Dataset | | | | Overlap-Weighted PSM Dataset | | | |
| --- | --- | --- | --- | --- | --- | --- | --- | --- |
|  | SCH (n=262) | MCH (n=133) | SMD | p-value | SCH (n=66.13) | MCH (n=66.13) | SMD | p-value |
| Awareness of the difference  between NIPV and non-NIPV | 124 (47.33) | 69 (51.88) | 0.091 | 0.454 | 32.57 (49.26) | 35.88 (54.27) | 0.100 | 0.416 |
| Non-NIPV importance | 206 (78.63) | 101 (75.94) | 0.064 | 0.632 | 52.48 (79.36) | 49.87 (75.41) | 0.094 | 0.442 |
| Non-NIPV safety | 183 (69.85) | 98 (73.68) | 0.085 | 0.498 | 45.56 (68.90) | 49.65 (75.08) | 0.138 | 0.268 |
| Effectiveness of non-NIPV | 175 (66.79) | 84 (63.16) | 0.076 | 0.544 | 44.18 (66.81) | 41.69 (63.05) | 0.079 | 0.518 |
| Concern SAE of non-NIPV | 203 (77.48) | 78 (58.65) | 0.412 | <0.001 | 50.39 (76.20) | 42.17 (63.77) | 0.274 | 0.028 |
| Price reasonableness  of non-NIPV | 93 (35.50) | 49 (36.84) | 0.028 | 0.879 | 24.76 (37.44) | 24.70 (37.35) | 0.002 | 0.988 |
| Healthcare professionals’ recommendations on non-NIPV | 256 (97.71) | 118 (88.72) | 0.363 | <0.001 | 63.96 (96.72) | 63.31 (95.74) | 0.051 | 0.644 |
| Child ever received non-NIPV | 125 (47.71) | 93 (69.92) | 0.463 | <0.001 | 34.82 (52.65) | 47.07 (71.17) | 0.389 | 0.002 |
| WNV in the future | 178 (67.94) | 78 (58.65) | 0.194 | 0.086 | 43.85 (66.31) | 38.76 (58.62) | 0.159 | 0.195 |

Abbreviations: PSM: Propensity Score Matching; SCH: Single-Child Households; MCH: Multiple-Children Households; SMD: Standardized Mean Difference; NIPV: National Immunization Program Vaccine; SAE: Serious Adverse Effects; WNV willingness to vaccinate with non-NIPV

Notes: Values in parentheses are percentages unless otherwise indicated.
